# Cognitive performance’s critical role in the progression from educational attainment to moderate to vigorous physical activity: insights from a Mendelian randomization study

**DOI:** 10.3389/fpsyg.2024.1421171

**Published:** 2024-07-05

**Authors:** Fang Qi, Zhang Jinmin

**Affiliations:** ^1^Chengdu Sport University, Chengdu, China; ^2^School of Physical Education and Sport Science, Fujian Normal University, Fuzhou, China

**Keywords:** cognitive performance, educational attainment, moderate to vigorous physical activity, Mendelian randomization, relationship

## Abstract

**Introduction:**

In individuals with high educational levels, moderate to vigorous physical activity (MVPA) is often elevated, yet the causal direction and the role of cognitive performance in this association remain ambiguous. Herein, Mendel randomization (MR) was employed to measure the causal relationship between education, cognitive performance, and moderate to vigorous physical activity. The purpose of this study was to analyze the causal effects of educational attainment on moderate-to-vigorous physical activity (MVPA) levels and to explore potential mediating factors.

**Methods:**

Two-sample univariate MR analysis was conducted to assess the overall effect of education on moderate to severe physical activity. Besides, a two-step MR analysis was carried out to evaluate the mediating effect of cognitive performance on the impact of education on moderate to severe physical activity. Individuals included were exclusively of European ancestry, with data gathered from extensive genome-wide association studies (GWAS) on education (*n* = 470,941), cognitive performance (*n* = 257,841), and moderate-to-vigorous physical activity (MVPA) (*n* = 377,234). Educational attainment was measured by college graduation status. Cognitive performance encompasses not only psycho-motor speed, memory, and abstract reasoning abilities but also knowledge and skills acquired in professional domains. MVPA is defined as any physical activity that produces a metabolic equivalent (MET) of ≥3.0.

**Results:**

The positive two-sample MR analysis showed that education level had a significant protective effect on MVPA deficiency (β = −0.276, 95% CI = −0.354 to −0.199, *p* = 2.866 × 10^−12^). However, the reverse two-sample MR analysis showed that MVPA had no significant causal relationship with education level (*p* = 0.165). Subsequently, the two-step MR analysis indicated that the potential causal protective effect of education on the risk of MVPA deficiency was mostly mediated by cognitive performance (mediating effect β = −0.235, 95% CI = −0.434 to −0.036, and the intermediary ratio was 85.061%).

**Discussion:**

Cognitive performance holds considerable significance in the relationship between education level and MVPA. Consequently, the intervention of cognitive performance may greatly improve the risk of physical inactivity caused by education, thereby promoting individual health.

## 1 Introduction

Regular moderate to severe PA (MVPA) can bring more additional health benefits, including reducing the risk of obesity, cardiovascular disease and degenerative symptoms (Mountjoy et al., 2011). As per the World Health Organization’s guidelines on physical activity (WHO, 2010), adults should aim for at least 150 min of moderate-intensity physical activity or 75 min of high-intensity physical activity per week, or an equivalent combination of both. However, in 2016, more than a quarter of the world’s adults were reported not to get enough physical activity. This left more than 1.4 billion adults vulnerable to developing or worsening diseases associated with physical inactivity, demanding urgent solutions (Guthold et al., 2018).

The level of education is usually measured by educational background or years of schooling. Education level (EA) is moderately heritable (Cutler and Lleras-Muney, 2006; Branigan et al., 2013), and has important correlation with health outcomes (Conti et al., 2010). For example, people with higher education tend to live longer (Mackenbach et al., 2008). Highly educated individuals often possess a better understanding of sports and health, leading them to actively engage in MVPA more frequently. However, there are also studies indicating a positive bivariate correlation between education level and sedentary behavior, and a negative correlation between education level and mild PA (Comes et al., 2019). Therefore, the causal relationship and potential mechanism between education level and physical activity are still unclear, leading to ongoing debates in the field.

Cognitive ability is an important predictor of education, health, and longevity (Lövdén et al., 2020), which usually includes fluid ability and crystallization ability (Baltes et al., 1999). On the one hand, cognitive performance is closely related to education level. In the general population, higher education level is related to higher neurocognitive test performance (Elliott et al., 2019). On the other hand, low cognitive performance often correlates with low levels of physical activity. Patients experiencing cognitive impairment typically engage in minimal physical activity and often struggle with adhering to exercise interventions. Severely mentally ill patients exhibit a notable decrease in strenuous exercise and an increase in sedentary behavior (Stubbs et al., 2016; Vancampfort et al., 2016). Therefore, cognitive performance seems to be a mediating factor in the causal relationship between education level and MVPA.

The recent large-scale genome-wide association study (GWAS) on education (*n* = 480,941), cognitive performance (*n* = 257,841) and MVPA (*n* = 377,234) offers an opportunity to clarify the complex causal relationship through MR analysis. This method uses genetic variation as a proxy for the phenotype of interest (i.e., instrumental variable) to assess causality. In contrast to observational studies, MR analysis benefits from the characteristics of heritable and randomly assigned genetic variation. This advantage allows MR analysis to mitigate potential confounding factors and reverse causal bias (Smith and Ebrahim, 2003; Zheng et al., 2017). The objective of this study is to apply the MR framework to investigate the impact of education and cognitive performance on MVPA levels. For cognitive performance factors that are supported by MR analysis to have a causal effect on MVPA, we aim to further employ MR mediation analysis to examine the extent to which these cognitive factors may mediate the influence of educational attainment. Our research hypotheses are twofold: (1) Educational attainment has a negative linear relationship with the risk of insufficient moderate-to-vigorous physical activity; and (2) Cognitive performance plays a crucial role in the pathway from educational attainment to MVPA. This study has important implications for designing targeted public health policies to improve MVPA and minimize health-related social disparities.

## 2 Method

**Figure 1:**
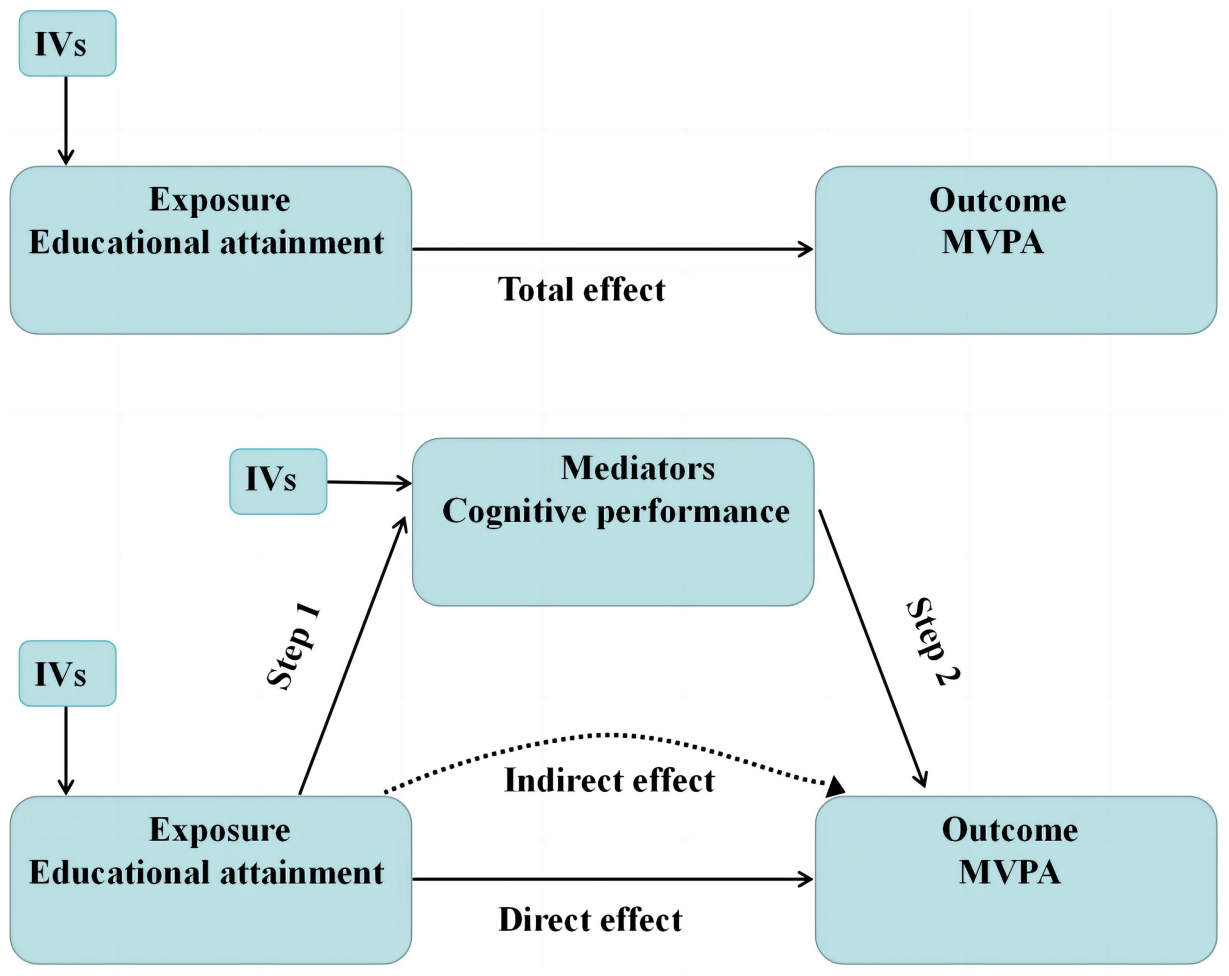
Graphical summary of the design of this study.

Herein, two-sample univariate and two-step MR analyses were employed to assess the causal relationship between education level, cognitive performance, and MVPA (Figure 1). Through univariate MR analysis, the total causal impact of education level on MVPA was evaluated. In addition, two-step MR analysis was carried out to assess the potential mediating role of cognitive performance between education level and MVPA. The GWAS data used in this study were hereby summarized, as shown in Table 1. The genetic association for educational attainment was estimated from a GWAS of 480,941 individuals of European descent (Loh et al., 2018). Educational attainment was harmonized across cohorts using the International Standard Classification of Education (Okbay et al., 2016). Genetic association estimates for cognitive performance were obtained from a GWAS conducted on 257,841 white individuals (Klimentidis et al., 2018). The measure of cognitive performance was the respondent’s score on a language-based cognitive test, specifically a cognitive test from the Wisconsin Longitudinal Study (WLS) that has excellent retest reliability and psychometric properties. Genetic association estimates for MVPA were obtained from a GWAS involving 377,234 white individuals (Lee et al., 2018). A large prospective cohort study from the UK Biobank included MVPA-related self-reported outcomes in a manner similar to the International Physical Activity Questionnaire, wrist-worn accelerometer variables, and genetic data. Full details of the GWAS analyses can be found in their original publications.

**Table 1:** Overview of GWAS data used in MR.

Phenotype	Number of participants	Ancestry	Author	Year of publication	Pubmed ID	Source (accessed on 11 April, 2024)
Educational attainment	470,941	European	Loh PR	2018	29,892,013	https://gwas.mrcieu.ac.uk/datasets/ebi-a-GCST90029012/
MVPA	377,234	European	Klimentidis YC	2018	29,899,525	https://gwas.mrcieu.ac.uk/datasets/ebi-a-GCST006097/
Cognitive performance	257,841	European	Lee JJ	2018	30,038,396	https://gwas.mrcieu.ac.uk/datasets/ebi-a-GCST006572/

### 2.1 Design

MR is a gene-based analysis method that leverages the random distribution of gene variation during pregnancy to infer the causal effect of exposure on the results. SNPs used as genetic tools in MR analysis must meet the following three basic assumptions: (1) Genetic variation must be truly related to exposure; (2) Genetic variation should not be associated with any confounding factors related to exposure outcome; (3) No direct correlation exists between genetic variation and results. Specifically, the inclusion criteria for the (1) relevance assumption: the inclusion criteria were SNPs strongly correlated with the exposure (statistical significance *p* < 5 × 10^–8^, linkage disequilibrium *r*^2^ < 0.001, strength statistic *F* > 10), while the exclusion criteria were SNPs not significantly or weakly correlated with the exposure factors; (2) independence assumption: the inclusion criteria were SNPs unrelated to confounders, and the exclusion criteria were SNPs associated with multiple confounders or known genetic factors influencing the outcome; (3) exclusion-restriction assumption: the inclusion criteria were SNPs unrelated to the outcome, and the exclusion criteria were SNPs related to the outcome. More details of this method have been previously described (Dimou and Tsilidis, 2018). The present study adhered to the STROBE-MR guidelines (Skrivankova et al., 2021). In addition, the present analysis lacked preview registration, so the results should be considered exploratory.

### 2.2 Data sources

Therefore, each exposure instrument considered in the univariable MR analyses was selected as variants associated with genome-wide significance (*p* < 5 × 10^–8^), linkage disequilibrium *r*^2^ < 0.001, and clustered variants distance >10,000 kb with the exposure. Additionally, to assess instrument strength, F statistics were calculated for each genetic instrument. In cases where the target SNP was unavailable in the result dataset, a proxy SNP with an R2 > 0.8 was used as a substitute. A total of 205 education level SNPs (Supplementary Table S1) were selected as genetic tools. Herein, the average F statistic of education level was 45.885 (ranging from 28.913 to 198.930) (Supplementary Table S2). Besides, a total of 19 MVPA SNPs (Supplementary Table S3) were selected as genetic tools. The average F statistic of MVPA was 34.393 (ranging from 29.984 to 51.824) (Supplementary Table S4). Additionally, 139 cognitive performance SNPs (Supplementary Table S5) were selected as genetic tools. The average F statistic of cognitive performance was 43.742 (ranging from 29.793 to 125.282) (Supplementary Table S6).

### 2.3 Preliminary analysis

In this study, a single-variable MR approach was employed. Specifically, a two-sample two-way MR analysis was conducted to evaluate the causal relationship between education level and MVPA. The instrumental variable for the exposure data, derived from the GWAS results, was extracted using the SNP tool. In order to ensure correct coordination of alleles, SNPs were targeted, so that the effect variants of exposure and results corresponded to the same allele (Hemani et al., 2018). The main result was generated using the inverse variance weighted (IVW) method of random effects. It should be noted that the IVW method provides unbiased estimates only when the assumption of balanced or non-existent pleiotropy holds (Hemani et al., 2018). Other MR methods included MR Egger, Weighted media, Weighted mode, and Simple mode.

For all significant MR results regarding the association between education level and physical activity, a two-step MR analysis was conducted to examine the potential mediating effect of cognitive performance. Two-step MR assesses whether intermediate traits act as mediators between exposure and outcome (Relton and Davey Smith, 2012). When exposure affected mediating variables and then impacted outcomes, the “coefficient product” was utilized (VanderWeele, 2016). Subsequently, the total effect of education level was decomposed into direct effects (i.e., the impact of education level on MVPA irrelevant to mediation) and indirect effect (i.e., the influence of education level on MVPA through mediation). Currently, this method has been extensively used (Burgess et al., 2015).

### 2.4 Sensitivity analysis

A genetic variation was deemed weak when the F-statistic was below 10. Such weakness might introduce deviations in the results (Palmer et al., 2011). In addition, multiple sensitivity analysis was conducted to verify the robustness of MR inspection, such as MR Egger intercept test, Cochran’s Q test, MR pleiotropic residual sum and leave-one-out analysis. Furthermore, all MR analyses were conducted using the TwoSampleMR package (version 0.5.8) in R (version 4.3.2). The original data and code files were stored in the Open Science Framework.^1^

### 2.5 Measuring the strength of evidence

Beta is the causal effect size we aim to estimate, i.e., the impact of the exposure factor on the outcome variable. CI is the confidence interval of the estimated parameter; usually, a 95% confidence interval is given to indicate the uncertainty of the estimated effect (Bland and Altman, 1996). The *p*-value is the *p*-value for testing the statistical hypothesis, used to evaluate whether the observed effect is statistically significant. In this study, the beta and its 95% CI and *p*-value data were all calculated and generated by the statistical software R. In this study, we not only use *p*-value thresholds to indicate statistical significance but also interpret the evidence provided by the results by examining the magnitude of the effect of interest and the width of its 95% confidence interval, combined with the consistency of the results from the different methods employed (Wasserstein et al., 2019).

## 3 Results

### 3.1 Causal effect of educational attainment on physical activity levels

Univariate positive MR analysis was hereby conducted to determine the protective effect of education level on MVPA deficiency. Higher education was found to be negatively correlated with lower MVPA (IVW: β = −0.276, 95%CI = −0.354 to −0.199, *p* = 2.866 × 10^−12^). Consistent univariate results were observed when using Weighted media, Simple mode, and Weighted mode analysis (Supplementary Figure S1; Supplementary Table S7). In all analyses, the MR Egger intercept was close to zero, and the 95% CI was narrow. There was indication of SNP effect heterogeneity, but none of the analyses pinpointed specific SNPs as drivers of the causal relationship (Supplementary Figure S2).

**Figure 2:**
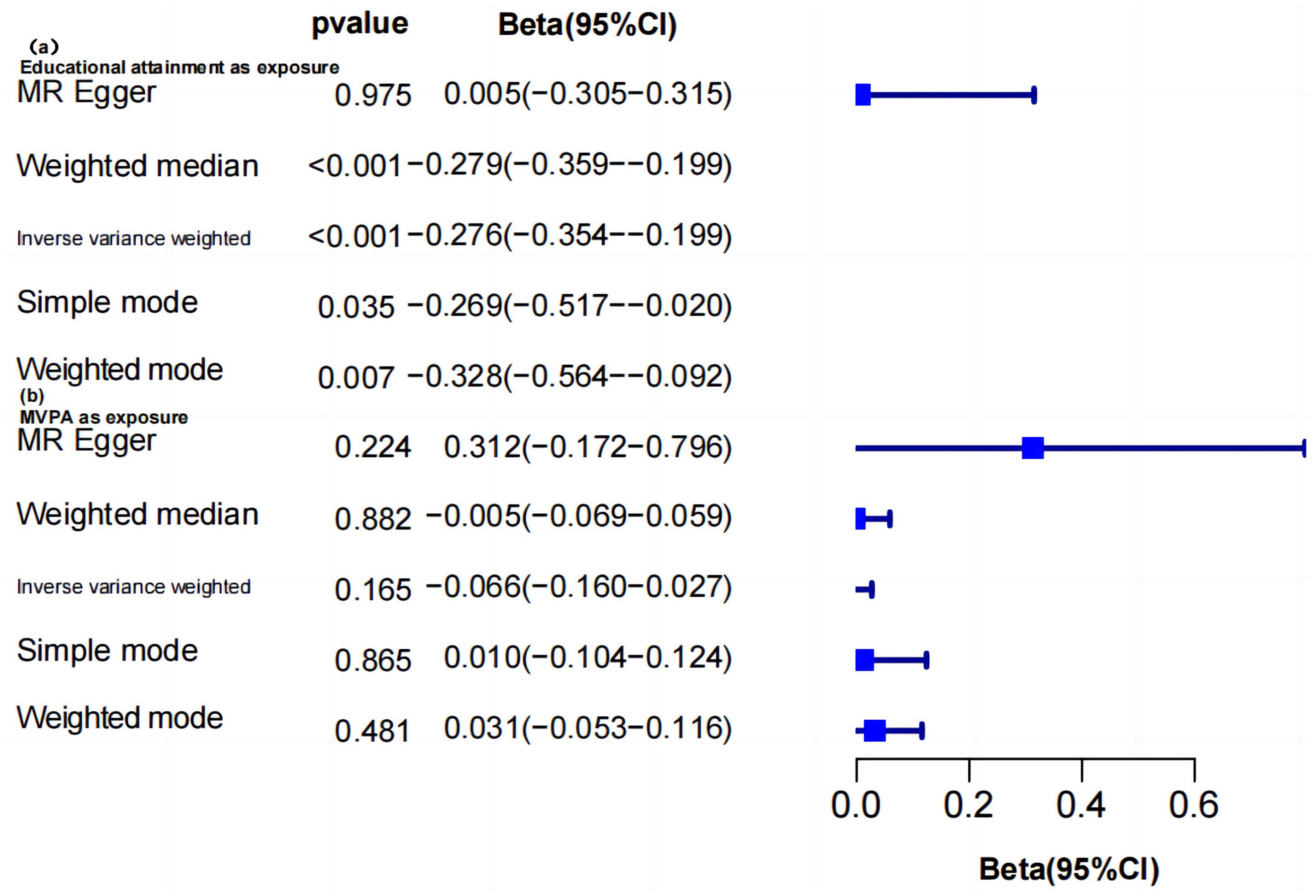
Two-way MR results between education level and MVPA.

The reverse MR analysis showed that MVPA did not affect educational attainment (IVW: *p* = 0.165). Consistent negative results were obtained using other MR methods (*p* > 0.05) (Supplementary Table S8). The MR Egger intercept of all analyses was close to zero, indicating that horizontal pleiotropy did not significantly impact the MR results. Besides, Cochran’s Q test showed the heterogeneity of SNP effect. However, one analysis left did not uncover any abnormal values (Supplementary Figure S3), and the funnel chart was roughly symmetrical on both sides (Supplementary Figure S4). The huge difference between univariate forward MR analysis and reverse MR analysis results (Figure 2) indicated that the causal effect of education on MVPA might depend on mediators.

### 3.2 Intermediary analysis

Through a two-step MR analysis, the causal link between education level and MVPA mediated by cognitive performance indicators was examined. Initially, education level was utilized as an instrumental variable to assess the causal relationship between the exposure and potential mediators. In terms of education level and mediation, a significant causal relationship was observed (Supplementary Table S9) (IVW: β = 1.639, 95%CI = 1.517 to 1.760, *p* = 3.198 × 10^−154^). Secondly, a relationship between cognitive performance and MVPA deficiency was identified by using genetic instruments of cognitive performance to assess the causal impact of mediators on MVPA deficiency (Supplementary Table S10) (IVW: β = −0.143, 95%CI = −0.175 to −0.111, *p* = 1.2 × 10^−18^). Mediation analysis showed that the impact of education level on MVPA was mostly mediated by cognitive performance, and the mediating effect of cognitive performance (β = −0.235, 95% CI = −0.434 to −0.036). Besides, the intermediary ratio was 85.061% (Table 2).

**Table 2:** Mediating effect of education level on MVPA’s cognitive performance.

Mediator	Total effect β (95% CI)	Direct effect A (95% CI)	Direct effect B (95% CI)	Mediation effect (95% CI)	Types of mediation	Mediator
Cognitive performance	−0.276 (−0.354 to −0.199)	1.639 (1.517 to 1.760)	−0.143 (−0.175 to −0.111)	−0.235 (−0.434 to −0.036)	Partial mediation	85.06%

“Direct effect A” indicates the influence of education level on cognitive performance; and “Direct effect B” indicates the influence of cognitive performance on MVPA.

## 4 Discussion

In this study, the causal impact of education on MVPA was explored using genetic variation as a non-confounding proxy univariate MR analysis. Through the application of five complementary univariate MR methods with distinct basic assumptions, the findings consistently indicated a positive correlation between higher education levels and a reduced risk of MVPA. Moreover, the reverse MR analysis demonstrated that MVPA had no causal effect on education level. Furthermore, given the significant contrast in the causal effects estimated by univariate forward MR analysis and reverse MR analysis, a two-step MR analysis was further conducted. This approach revealed that the influence of education level on the risk of MVPA deficiency primarily stemmed from the protective effect of cognitive performance, with education level playing a partial role. Our work leverages large-scale GWAS data to investigate the effects of genetically predicted educational attainment and cognitive performance on MVPA risk within the MR framework, providing evidence supporting the protective roles of education and cognitive performance. Following extensive sensitivity analyses and multiple validity assessments, the results remained robust.

Our MR analyses identified a protective effect of genetically predicted educational attainment on MVPA risk. Our findings align with previously published research conclusions. Specifically, elementary schools often adopt recess, classroom PA breaks, before- and after-school programs, and integration of PA with academic lessons to create healthy school environments that promote students reaching recommended MVPA levels (Committee on Physical Activity and Physical Education in the School Environment et al., 2013). Higher education institutions typically provide more supportive environments for promoting students meeting MVPA guidelines, such as highly professional physical education faculty, rich and diverse sports club activities, and sufficient advanced sports facilities (Carlson et al., 2014). Educational attainment may also influence individuals’ socioeconomic resources and environmental exposures in adulthood, directly or indirectly determining access to quality healthcare, including exercise prescription interventions to achieve MVPA recommendations (Ceci, 1991). Further research indicates that parental education level in the family can impact children’s MVPA frequency (Jiménez-Pavón et al., 2012). Parents support their children’s MVPA levels by indirectly encouraging or directly taking them to engage in physical exercise (Gustafson and Rhodes, 2006).

On the other hand, our current work also improves upon some previous studies. Specifically, increasing evidence from behavioral research and neuroscience suggests that motor and cognitive development are inherently intertwined (Zhou and Tolmie, 2024). Physical activity has been found to be significantly associated with cognitive performance in children and adolescents (Zhang et al., 2022). Furthermore, our MR study explores the negative correlation between cognitive performance and MVPA risk, as we employ more genetic instruments for cognitive performance and provide greater robustness against potential violations of MR modeling assumptions. The protective effect of cognitive performance on MVPA risk may be attributed to good cognitive performance assisting individuals in adopting and adhering to healthy behaviors, as well as increasing awareness of the detrimental effects of sedentary behavior and knowledge about the health benefits of MVPA (Bor et al., 2017).

The association between education and cognitive ability is evident across the adult lifespan and the entire educational spectrum. Evidence suggests that cognitive ability is related to the choice to pursue longer education, particularly higher education, with higher educational attainment correlating with higher baseline cognitive levels (Lövdén et al., 2020). Concurrently, compared to individuals with lower educational attainment, those with higher education have a reduced risk of dementia and Alzheimer’s disease (AD) in old age (Karp et al., 2004; Caamaño-Isorna et al., 2006). Consistent with these results, our MR study reports a positive impact of education on cognitive function. A possible explanation is that education may influence cognitive ability development through a broad foundation of specific knowledge and skills, such as improvements in memory, reasoning, cognitive strategies, and test-taking abilities. It is well known that educational attainment is heritable, and cognitive performance is considered a downstream regulatory factor of education. Thus, our evidence supports that the protective effect of education on MVPA risk is primarily mediated through cognitive performance, with the underlying mechanism potentially related to the exceptional self-management awareness and exercise health participation practices provided to highly educated individuals.

Therefore, it was hereby proposed that schools held an indisputable responsibility for fostering the healthy development of students. This involved instilling a cognitive understanding of health and promoting a correct comprehension of MVPA. Additionally, implementing MVPA interventions was essential to assist students in achieving the recommended levels and reaping the benefits of MVPA. The correct cognition in student days and the habit of regular MVPA exercise could facilitate individuals to maintain a healthy lifestyle in future life. As students transition into society, it is vital for society to offer necessary support, including fostering a positive social sports atmosphere, providing ample sports opportunities, and ensuring top-notch sports infrastructure. Ultimately, individuals must translate their understanding of the importance of MVPA into daily behavior and maintain consistent practice in their everyday lives.

## 5 Advantages and limitations

This study made the first attempt to comprehensively investigate the causal relationship between education level, cognitive performance, and MVPA using the MR method, serving as a genetic tool selected from the GWAS of the largest and latest education level, cognitive performance, and MVPA. Under the univariate MR design, a series of sensitivity analyses were conducted to control the bias of multiple validity and verify the robustness of MR results. This research, employing genetic tools, unveiled the predominant causal link between cognitive performance and the relationship between education level and MVPA. Overall, the discovery offers fresh insights into the social disparities in MVPA behavior and health inequality.

However, the research results still have some limitations to be considered when interpreting the results. First, there was evidence of heterogeneity implied by Cochran Q statistics. To solve this problem, the IVW random effects method was chosen as the main MR method, which was robust to heterogeneity. Secondly, in the MR analysis of two samples, sample overlap might introduce potential deviation. Since most of the genetic variations used in the analysis were strong IV, F statistic > 28.913, and were related to *p* < 5 × 10 ^−8^ exposure, any deviations from the expected outcomes were expected to be minimal. Finally, to address population stratification, the present analysis focused solely on individuals of European descent. However, further research involving other racial groups is still warranted to ensure broader applicability of the findings.

Therefore, it was hereby proposed that schools held an indisputable responsibility for fostering the healthy development of students. This involved instilling a cognitive understanding of health and promoting a correct comprehension of MVPA. Additionally, implementing MVPA interventions was essential to assist students in achieving the recommended levels and reaping the benefits of MVPA. The correct cognition in student days and the habit of regular MVPA exercise could facilitate individuals to maintain a healthy lifestyle in future life. As students transition into society, it is vital for society to offer necessary support, including fostering a positive social sports atmosphere, providing ample sports opportunities, and ensuring top-notch sports infrastructure. Ultimately, individuals must translate their understanding of the importance of MVPA into daily behavior and maintain consistent practice in their everyday lives. Our research findings support the potential causal protective effect of educational attainment on MVPA risk, which is mediated by cognitive performance. Therefore, educational level and cognitive functioning may serve as public health targets for reducing the risk of MVPA.

## 6 Conclusion

This study utilizes genetic data from MR analysis to generate evidence supporting the protective role of education, with evidence indicating that the impact of education is primarily mediated through cognitive performance. One plausible explanation for the crucial role of cognitive functioning is its potential to exert widespread beneficial effects on health. These findings underscore the importance of educational attainment and cognitive performance as partial determinants of MVPA risk, which may serve as potential targets for reducing health inequalities stemming from MVPA risk.

## Data availability statement

The original contributions presented in the study are included in the article/Supplementary material, further inquiries can be directed to the corresponding author.

## Ethics statement

The GWAS data utilized in this study were sourced from the UK Biobank, a publicly accessible resource available for download by qualified researchers. Prior to participation, all subjects provided written, informed consent. Ethical approval for the UK Biobank study was granted by the North West Multi-Centre Research Ethics Committee, the National Information Governance Board for Health and Social Care (NIGB), and the Community Health Index Advisory Group (CHIAG), authorizing research involving human participants.

## Author contributions

FQ: Writing – review & editing. ZJ: Writing – original draft.

## Footnotes

^1^ https://osf.io/crwah/

## References

[ref1] Baltes P. B. Staudinger U. M. Lindenberger U. (1999). Lifespan psychology: theory and application to intellectual functioning. Annu. Rev. Psychol. 50, 471–507. doi: 10.1146/annurev.psych.50.1.471, PMID: 15012462

[ref2] Bland J. M. Altman D. G. (1996). Statistics notes: transformations, means, and confidence intervals. BMJ 312:1079. doi: 10.1136/bmj.312.7038.1079, PMID: 8616417 PMC2350916

[ref3] Bor J. Cohen G. H. Galea S. (2017). Population health in an era of rising income inequality: USA, 1980-2015. Lancet 389, 1475–1490. doi: 10.1016/s0140-6736(17)30571-8 28402829

[ref4] Branigan A. R. McCallum K. J. Freese J. (2013). Variation in the heritability of educational attainment: an international Meta-analysis. Soc. Forces 92, 109–140. doi: 10.1093/sf/sot076

[ref5] Burgess S. Daniel R. M. Butterworth A. S. Thompson S. G. (2015). Network Mendelian randomization: using genetic variants as instrumental variables to investigate mediation in causal pathways. Int. J. Epidemiol. 44, 484–495. doi: 10.1093/ije/dyu176, PMID: 25150977 PMC4469795

[ref6] Caamaño-Isorna F. Corral M. Montes-Martínez A. Takkouche B. (2006). Education and dementia: a meta-analytic study. Neuroepidemiology 26, 226–232. doi: 10.1159/000093378, PMID: 16707907

[ref7] Carlson J. A. Mignano A. M. Norman G. J. McKenzie T. L. Kerr J. Arredondo E. M.. (2014). Socioeconomic disparities in elementary school practices and children's physical activity during school. Am. J. Health Promot. 28, S47–S53. doi: 10.4278/ajhp.130430-QUAN-206, PMID: 24380465 PMC4082956

[ref8] Ceci S. J. (1991). How much does schooling influence general intelligence and its cognitive components? A reassessment of the evidence. Dev. Psychol. 27, 703–722. doi: 10.1037/0012-1649.27.5.703

[ref9] Comes A. L. Senner F. Budde M. Adorjan K. Anderson-Schmidt H. Andlauer T. F. M.. (2019). The genetic relationship between educational attainment and cognitive performance in major psychiatric disorders. Transl. Psychiatry 9:210. doi: 10.1038/s41398-019-0547-x, PMID: 31462630 PMC6713703

[ref10] Committee on Physical Activity and Physical Education in the School Environment (2013) in Educating the student body: Taking physical activity and physical education to school. eds. Kohl H. W. III Cook H. D. (Washington, DC: National Academies Press). 24851299

[ref11] Conti G. Heckman J. Urzua S. (2010). The education-health gradient. Am. Econ. Rev. 100, 234–238. doi: 10.1257/aer.100.2.234, PMID: 24741117 PMC3985402

[ref12] Cutler D. Lleras-Muney A. (2006). “Education and health: Evaluating theories and evidence” in Paper presented at the Health Effects of Non-Health Policies (Cambridge, MA).

[ref13] Dimou N. L. Tsilidis K. K. (2018). A primer in Mendelian randomization methodology with a focus on utilizing published summary association data. Methods Mol. Biol. 1793, 211–230. doi: 10.1007/978-1-4939-7868-7_13, PMID: 29876899

[ref14] Elliott M. L. Belsky D. W. Anderson K. Corcoran D. L. Ge T. Knodt A.. (2019). A polygenic score for higher educational attainment is associated with larger brains. Cereb. Cortex 29, 3496–3504. doi: 10.1093/cercor/bhy219, PMID: 30215680 PMC6645179

[ref15] Gustafson S. L. Rhodes R. E. (2006). Parental correlates of physical activity in children and early adolescents. Sports Med. 36, 79–97. doi: 10.2165/00007256-200636010-00006 16445312

[ref16] Guthold R. Stevens G. A. Riley L. M. Bull F. C. (2018). Worldwide trends in insufficient physical activity from 2001 to 2016: a pooled analysis of 358 population-based surveys with 1·9 million participants. Lancet Glob. Health 6, e1077–e1086. doi: 10.1016/s2214-109x(18)30357-7, PMID: 30193830

[ref17] Hemani G. Zheng J. Elsworth B. Wade K. H. Haberland V. Baird D.. (2018). The MR-base platform supports systematic causal inference across the human phenome. eLife 7:e34408. doi: 10.7554/eLife.34408, PMID: 29846171 PMC5976434

[ref18] Jiménez-Pavón D. Fernández-Alvira J. M. te Velde S. J. Brug J. Bere E. Jan N.. (2012). Associations of parental education and parental physical activity (PA) with children's PA: the ENERGY cross-sectional study. Prev. Med. 55, 310–314. doi: 10.1016/j.ypmed.2012.07.011, PMID: 22846500

[ref19] Karp A. Kåreholt I. Qiu C. Bellander T. Winblad B. Fratiglioni L. (2004). Relation of education and occupation-based socioeconomic status to incident Alzheimer's disease. Am. J. Epidemiol. 159, 175–183. doi: 10.1093/aje/kwh018, PMID: 14718220

[ref20] Klimentidis Y. C. Raichlen D. A. Bea J. Garcia D. O. Wineinger N. E. Mandarino L. J.. (2018). Genome-wide association study of habitual physical activity in over 377,000 UK biobank participants identifies multiple variants including CADM2 and APOE. Int. J. Obes. 42, 1161–1176. doi: 10.1038/s41366-018-0120-3, PMID: 29899525 PMC6195860

[ref21] Lee J. J. Wedow R. Okbay A. Kong E. Maghzian O. Zacher M.. (2018). Gene discovery and polygenic prediction from a genome-wide association study of educational attainment in 1.1 million individuals. Nat. Genet. 50, 1112–1121. doi: 10.1038/s41588-018-0147-3, PMID: 30038396 PMC6393768

[ref22] Loh P. R. Kichaev G. Gazal S. Schoech A. P. Price A. L. (2018). Mixed-model association for biobank-scale datasets. Nat. Genet. 50, 906–908. doi: 10.1038/s41588-018-0144-6, PMID: 29892013 PMC6309610

[ref23] Lövdén M. Fratiglioni L. Glymour M. M. Lindenberger U. Tucker-Drob E. M. (2020). Education and cognitive functioning across the life span. Psychol. Sci. Public Interest 21, 6–41. doi: 10.1177/1529100620920576 32772803 PMC7425377

[ref24] Mackenbach J. P. Stirbu I. Roskam A. J. Schaap M. M. Menvielle G. Leinsalu M.. (2008). Socioeconomic inequalities in health in 22 European countries. N. Engl. J. Med. 358, 2468–2481. doi: 10.1056/NEJMsa0707519, PMID: 18525043

[ref25] Mountjoy M. Andersen L. B. Armstrong N. Biddle S. Boreham C. Bedenbeck H. P.. (2011). International Olympic Committee consensus statement on the health and fitness of young people through physical activity and sport. Br. J. Sports Med. 45, 839–848. doi: 10.1136/bjsports-2011-090228, PMID: 21836168

[ref26] Okbay A. Beauchamp J. P. Fontana M. A. Lee J. J. Pers T. H. Rietveld C. A.. (2016). Genome-wide association study identifies 74 loci associated with educational attainment. Nature 533, 539–542. doi: 10.1038/nature17671 27225129 PMC4883595

[ref27] Palmer T. M. Lawlor D. A. Harbord R. M. Sheehan N. A. Tobias J. H. Timpson N. J.. (2011). Using multiple genetic variants as instrumental variables for modifiable risk factors. Stat. Methods Med. Res. 21, 223–242. doi: 10.1177/0962280210394459, PMID: 21216802 PMC3917707

[ref28] Relton C. L. Davey Smith G. (2012). Two-step epigenetic Mendelian randomization: a strategy for establishing the causal role of epigenetic processes in pathways to disease. Int. J. Epidemiol. 41, 161–176. doi: 10.1093/ije/dyr233, PMID: 22422451 PMC3304531

[ref29] Skrivankova V. W. Richmond R. C. Woolf B. A. R. Yarmolinsky J. Davies N. M. Swanson S. A.. (2021). Strengthening the reporting of observational studies in epidemiology using Mendelian randomization: the STROBE-MR statement. JAMA 326, 1614–1621. doi: 10.1001/jama.2021.18236, PMID: 34698778

[ref30] Smith G. D. Ebrahim S. (2003). 'Mendelian randomization': can genetic epidemiology contribute to understanding environmental determinants of disease? Int. J. Epidemiol. 32, 1–22. doi: 10.1093/ije/dyg070 12689998

[ref31] Stubbs B. Firth J. Berry A. Schuch F. B. Rosenbaum S. Gaughran F.. (2016). How much physical activity do people with schizophrenia engage in? A systematic review, comparative meta-analysis and meta-regression. Schizophr. Res. 176, 431–440. doi: 10.1016/j.schres.2016.05.017, PMID: 27261419

[ref32] Vancampfort D. Firth J. Schuch F. Rosenbaum S. De Hert M. Mugisha J.. (2016). Physical activity and sedentary behavior in people with bipolar disorder: a systematic review and meta-analysis. J. Affect. Disord. 201, 145–152. doi: 10.1016/j.jad.2016.05.020, PMID: 27235817

[ref33] VanderWeele T. J. (2016). Mediation analysis: a Practitioner's guide. Annu. Rev. Public Health 37, 17–32. doi: 10.1146/annurev-publhealth-032315-021402, PMID: 26653405

[ref34] Wasserstein R. L. Schirm A. L. Lazar N. A. (2019). Moving to a world beyond “p < 0.05”. Am. Stat. 73, 1–19. doi: 10.1080/00031305.2019.1583913

[ref35] WHO (2010). Available at: https://www.who.int/publications/i/item/9789241599979 (Accessed April 21, 2024).

[ref36] Zhang D. Hong J. Chen S. Liu Y. (2022). Associations of physical activity with academic achievement and academic burden in Chinese children and adolescents: do gender and school grade matter? BMC Public Health 22:1496. doi: 10.1186/s12889-022-13886-3 35932047 PMC9356485

[ref37] Zheng J. Baird D. Borges M. C. Bowden J. Hemani G. Haycock P.. (2017). Recent developments in Mendelian randomization studies. Curr. Epidemiol. Rep. 4, 330–345. doi: 10.1007/s40471-017-0128-6, PMID: 29226067 PMC5711966

[ref38] Zhou Y. Tolmie A. (2024). Associations between gross and fine motor skills, physical activity, executive function, and academic achievement: longitudinal findings from the UK millennium Cohort Study. Brain Sci. 14:121. doi: 10.3390/brainsci14020121, PMID: 38391696 PMC10887312

