# Supplementary material for: Cognitive performance’s critical role in the progression from educational attainment to moderate to vigorous physical activity: insights from a Mendelian randomization study
Source: Front Psychol. 2024 Jul 5;15:1421171. doi: 10.3389/fpsyg.2024.1421171 (PMC11258795; doi:10.3389/fpsyg.2024.1421171)
Supplement: Supplementary file 1 [file Table_1.docx]

Supplementary Material

# Supplementary Figures


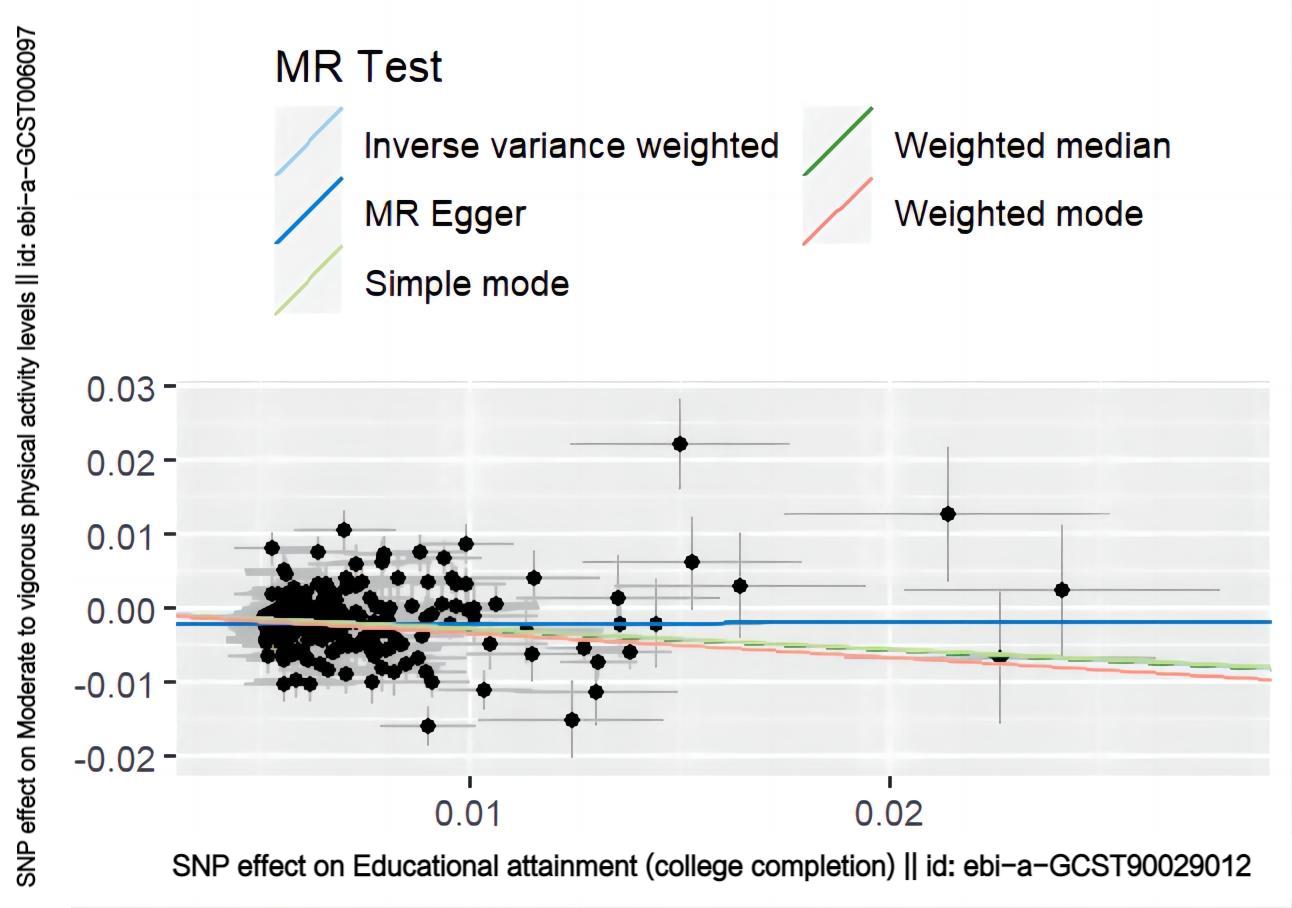


**Supplementary Figure 1**. Scatter plot of education attainment on moderate to vigorous physical activity levels


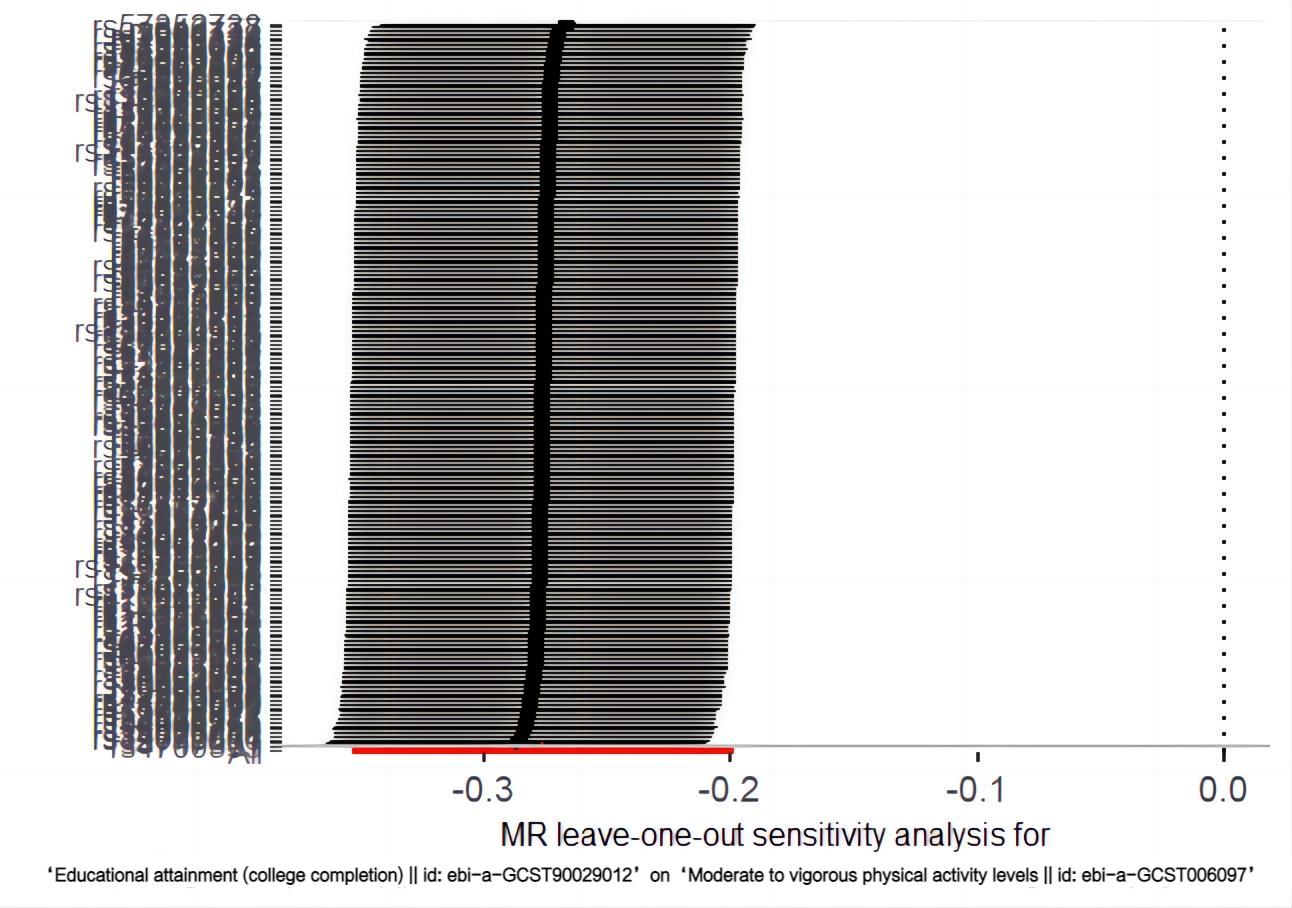


**Supplementary Figure 2**. MR leave-one-out sensitivity analysis for educational attainment on moderate to vigorous physical activity levels


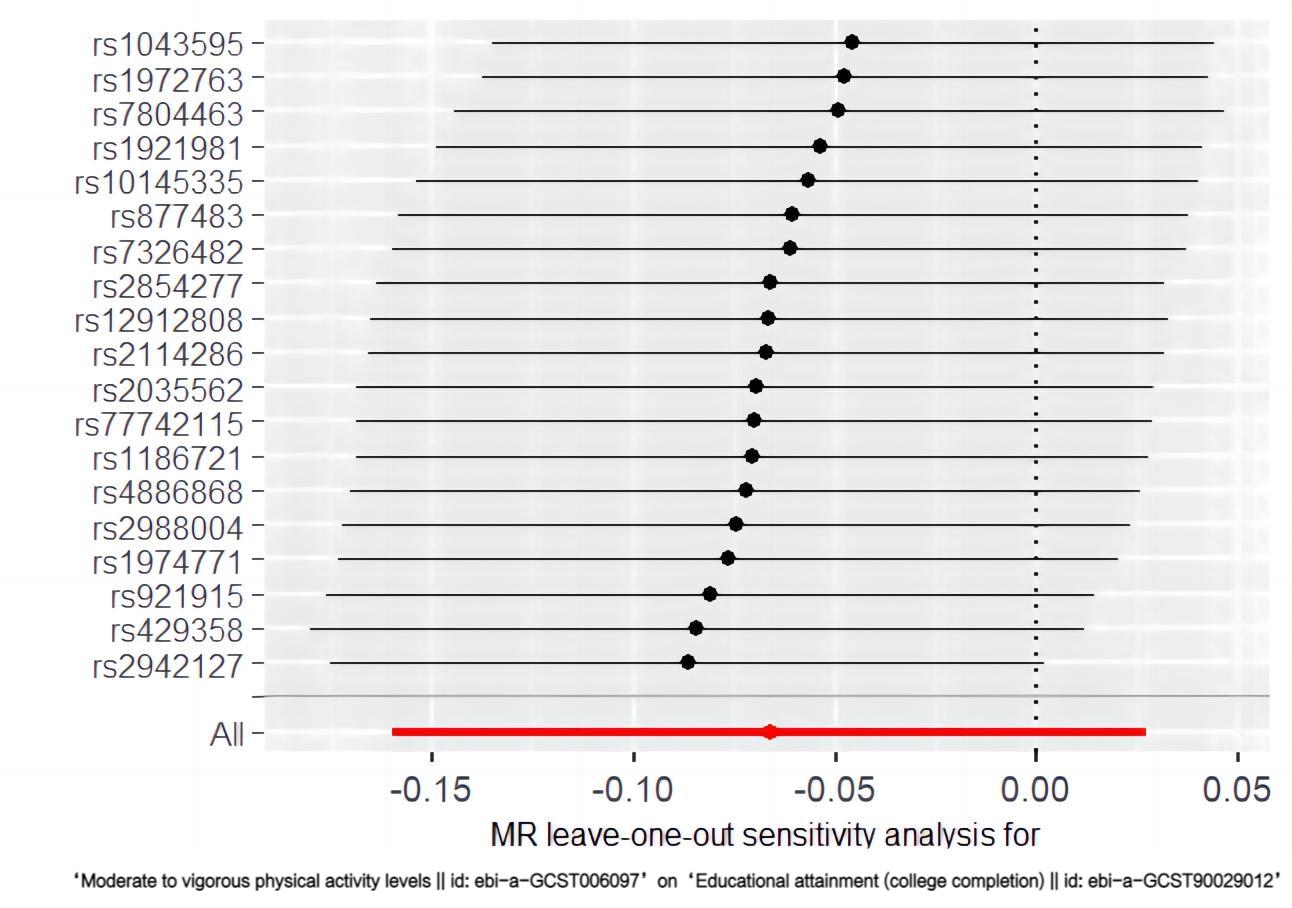


**Supplementary Figure 3**. MR leave-one-out sensitivity analysis for moderate to vigorous physical activity levels on educational attainment


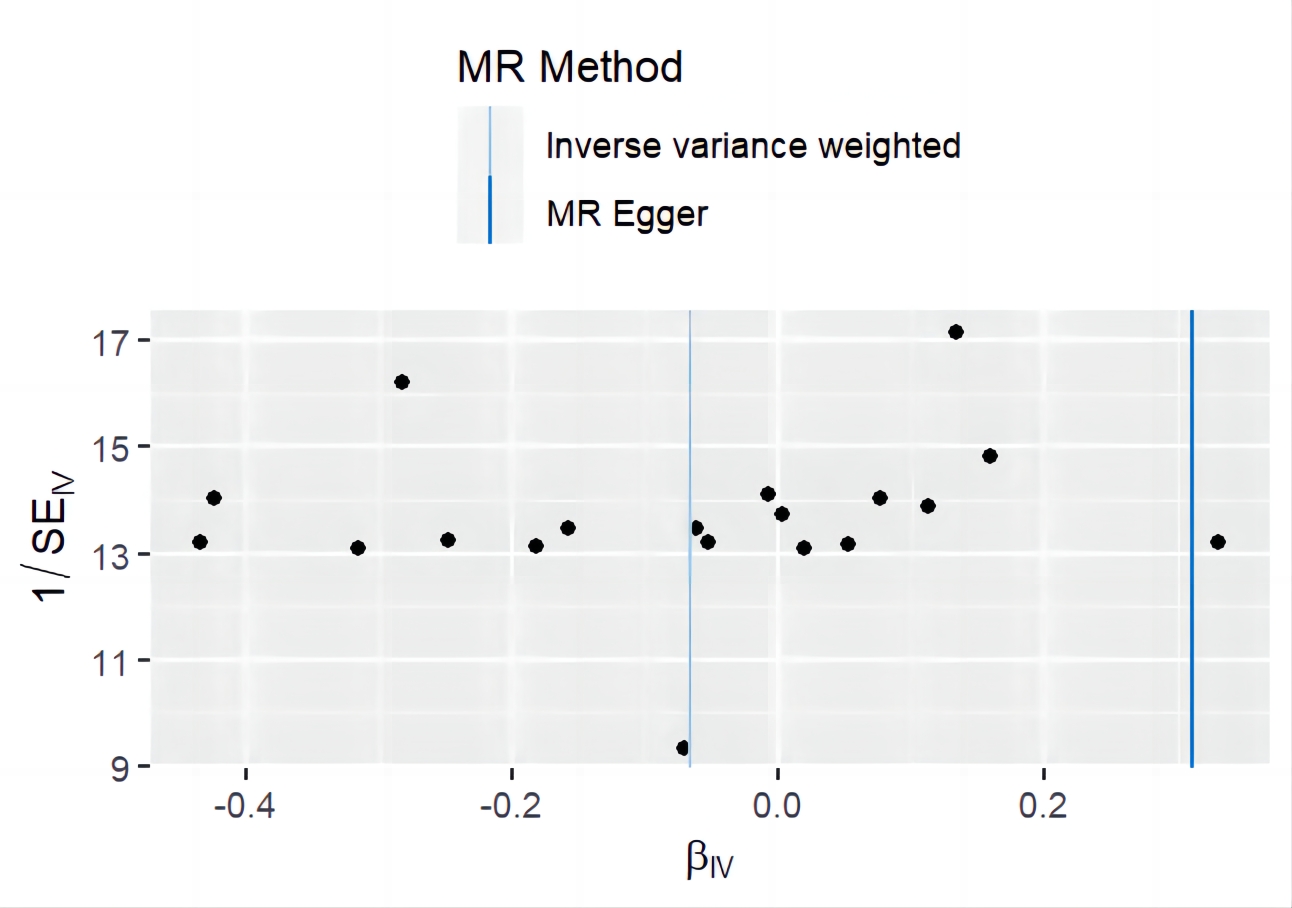


**Supplementary Figure 4**. Funnel plot of moderate to vigorous physical activity levels on educational attainment
