# Supplementary material for: Cognitive performance’s critical role in the progression from educational attainment to moderate to vigorous physical activity: insights from a Mendelian randomization study
Source: Front Psychol. 2024 Jul 5;15:1421171. doi: 10.3389/fpsyg.2024.1421171 (PMC11258795; doi:10.3389/fpsyg.2024.1421171)
Supplement: Supplementary file 2 [file Table_2.docx]

**STROBE-MR checklist of recommended items to address in reports of Mendelian randomization studies**^1^ ^2^

| **Item No.** | **Section** | **Checklist item** | **Page No.** | **Relevant text from manuscript** |
| --- | --- | --- | --- | --- |
| 1 | **TITLE and ABSTRACT** | Indicate Mendelian randomization (MR) as the study’s design in the title and/or the abstract if that is a main purpose of the study |  | Cognitive Performance's Critical Role in the Progression from Educational Attainment to Moderate to Vigorous Physical Activity: Insights from a Mendelian Randomization Study |
|  | **INTRODUCTION** |  |  |  |
| 2 | **Background** | Explain the scientific background and rationale for the reported study. What is the exposure? Is a potential causal relationship between exposure and outcome plausible? Justify why MR is a helpful method to address the study question |  | This advantage allows MR analysis to mitigate potential confounding factors and reverse causal bias (Smith and Ebrahim, 2003; Zheng et al., 2017). |
| 3 | **Objectives** | State specific objectives clearly, including pre-specified causal hypotheses (if any). State that MR is a method that, under specific assumptions, intends to estimate causal effects |  | The objective of this study is to apply the MR framework to investigate the impact of education and cognitive performance on MVPA levels. For cognitive performance factors that are supported by MR analysis to have a causal effect on MVPA, we aim to further employ MR mediation analysis to examine the extent to which these cognitive factors may mediate the influence of educational attainment. Our research hypotheses are twofold: (1) Educational attainment has a negative linear relationship with the risk of insufficient moderate-to-vigorous physical activity; and (2) Cognitive performance plays a crucial role in the pathway from educational attainment to MVPA. This study has important implications for designing targeted public health policies to improve MVPA and minimize health-related social disparities. |
|  | **METHODS** |  |  |  |
| 4 | **Study design and data sources** | Present key elements of the study design early in the article. Consider including a table listing sources of data for all phases of the study. For each data source contributing to the analysis, describe the following: |  |  |
|  | a) | Setting: Describe the study design and the underlying population, if possible. Describe the setting, locations, and relevant dates, including periods of recruitment, exposure, follow-up, and data collection, when available. |  | Herein, two-sample univariate and two-step MR analyses were employed to assess the causal relationship between education level, cognitive performance, and MVPA (Figure 1). Through univariate MR analysis, the total causal impact of education level on MVPA was evaluated. In addition, two-step MR analysis was carried out to assess the potential mediating role of cognitive performance between education level and MVPA. |
|  | b) | Participants: Give the eligibility criteria, and the sources and methods of selection of participants. Report the sample size, and whether any power or sample size calculations were carried out prior to the main analysis |  | The GWAS data used in this study were hereby summarized, as shown in Table 1.  The genetic association for educational attainment was estimated from a GWAS of 480,941 individuals of European descent(Loh et al., 2018).  Genetic association estimates for cognitive performance were obtained from a GWAS conducted on 257,841 white individuals (Klimentidis et al., 2018).  Genetic association estimates for MVPA were obtained from a GWAS involving 377,234 white individuals( Lee et al., 2018). |
|  | c) | Describe measurement, quality control and selection of genetic variants |  | Specifically, the inclusion criteria for the (1) relevance assumption: the inclusion criteria were SNPs strongly correlated with the exposure (statistical significance P < 5 × 10-8, linkage disequilibrium r² < 0.001, strength statistic F > 10), while the exclusion criteria were SNPs not significantly or weakly correlated with the exposure factors; (2) independence assumption: the inclusion criteria were SNPs unrelated to confounders, and the exclusion criteria were SNPs associated with multiple confounders or known genetic factors influencing the outcome; (3) exclusion-restriction assumption: the inclusion criteria were SNPs unrelated to the outcome, and the exclusion criteria were SNPs related to the outcome. |
|  | d) | For each exposure, outcome, and other relevant variables, describe methods of assessment and diagnostic criteria for diseases |  | Educational attainment was harmonized across cohorts using the International Standard Classification of Education (Okbay et al., 2016).  The measure of cognitive performance was the respondent's score on a language-based cognitive test.  MVPA-related self-reported outcomes in a manner similar to the International Physical Activity Questionnaire, wrist-worn accelerometer variables, and genetic data. |
|  | e) | Provide details of ethics committee approval and participant informed consent, if relevant |  | Ethic statement was claimed in the involving GWAS studies. |
| 5 | **Assumptions** | Explicitly state the three core IV assumptions for the main analysis (relevance, independence and exclusion restriction) as well assumptions for any additional or sensitivity analysis |  | SNPs used as genetic tools in MR analysis must meet the following three basic assumptions: (1) Genetic variation must be truly related to exposure; (2) Genetic variation should not be associated with any confounding factors related to exposure outcome; (3) No direct correlation exists between genetic variation and results. |
| 6 | **Statistical methods: main analysis** | Describe statistical methods and statistics used |  |  |
|  | a) | Describe how quantitative variables were handled in the analyses (i.e., scale, units, model) |  | Therefore, each exposure instrument considered in the univariable MR analyses was selected as variants associated with genome-wide significance (P < 5 × 10-8), linkage disequilibrium r2 < 0.001, and clustered variants distance > 10,000 kb with the exposure. |
|  | b) | Describe how genetic variants were handled in the analyses and, if applicable, how their weights were selected |  | Additionally, to assess instrument strength, F statistics were calculated for each genetic instrument. |
|  | c) | Describe the MR estimator (e.g. two-stage least squares, Wald ratio) and related statistics. Detail the included covariates and, in case of two-sample MR, whether the same covariate set was used for adjustment in the two samples |  | The main result was generated using the inverse variance weighted (IVW) method of random effects. It should be noted that the IVW method provides unbiased estimates only when the assumption of balanced or non-existent pleiotropy holds. (Hemani et al., 2018). Other MR methods included MR Egger, Weighted media, Weighted mode, and Simple mode. |
|  | d) | Explain how missing data were addressed |  | In cases where the target SNP was unavailable in the result dataset, a proxy SNP with an R2>0.8 was used as a substitute. |
|  | e) | If applicable, indicate how multiple testing was addressed |  | NA |
| 7 | **Assessment of assumptions** | Describe any methods or prior knowledge used to assess the assumptions or justify their validity |  | In this study, we not only use P-value thresholds to indicate statistical significance but also interpret the evidence provided by the results by examining the magnitude of the effect of interest and the width of its 95% confidence interval, combined with the consistency of the results from the different methods employed (Wasserstein et al., 2019). |
| 8 | **Sensitivity analyses and additional analyses** | Describe any sensitivity analyses or additional analyses performed (e.g. comparison of effect estimates from different approaches, independent replication, bias analytic techniques, validation of instruments, simulations) |  | In addition, multiple sensitivity analysis was conducted to verify the robustness of MR inspection, such as MR Egger intercept test, Cochran's Q test, MR pleiotropic residual sum and leave-one-out analysis. |
| 9 | **Software and pre-registration** |  |  |  |
|  | a) | Name statistical software and package(s), including version and settings used |  | Furthermore, all MR analyses were conducted using the TwoSampleMR package (version 0.5.8) in R (version 4.3.2). |
|  | b) | State whether the study protocol and details were pre-registered (as well as when and where) |  | In addition, the present analysis lacked preview registration, so the results should be considered exploratory. |
|  | **RESULTS** |  |  |  |
| 10 | **Descriptive data** |  |  |  |
|  | a) | Report the numbers of individuals at each stage of included studies and reasons for exclusion. Consider use of a flow diagram |  | educational attainment (n=470,941), cognitive performance (n=257,841), and MVPA (n=377,234) |
|  | b) | Report summary statistics for phenotypic exposure(s), outcome(s), and other relevant variables (e.g. means, SDs, proportions) |  | Supplementary Table S1  Supplementary Table S3  Supplementary Table S5 |
|  | c) | If the data sources include meta-analyses of previous studies, provide the assessments of heterogeneity across these studies |  | NA |
|  | d) | For two-sample MR:  i.  Provide justification of the similarity of the genetic variant-exposure associations between the exposure and outcome samples  ii.  Provide information on the number of individuals who overlap between the exposure and outcome studies |  | Since most of the genetic variations used in the analysis were strong IV, F statistic>28.913, and were related to P<5 × 10 -8 exposure, any deviations from the expected outcomes were expected to be minimal. |
| 11 | **Main results** |  |  |  |
|  | a) | Report the associations between genetic variant and exposure, and between genetic variant and outcome, preferably on an interpretable scale |  | Our work leverages large-scale GWAS data to investigate the effects of genetically predicted educational attainment and cognitive performance on MVPA risk within the MR framework, providing evidence supporting the protective roles of education and cognitive performance. Following extensive sensitivity analyses and multiple validity assessments, the results remained robust. |
|  | b) | Report MR estimates of the relationship between exposure and outcome, and the measures of uncertainty from the MR analysis, on an interpretable scale, such as odds ratio or relative risk per SD difference |  | Mediation analysis showed that the impact of education level on MVPA was mostly mediated by cognitive performance, and the mediating effect of cognitive performance (β=- 0.235,95% CI=-0.434 to -0.036). |
|  | c) | If relevant, consider translating estimates of relative risk into absolute risk for a meaningful time period |  | Besides, the intermediary ratio was 85.061% (Table 2). |
|  | d) | Consider plots to visualize results (e.g. forest plot, scatterplot of associations between genetic variants and outcome versus between genetic variants and exposure) |  | Figure 2 |
| 12 | **Assessment of assumptions** |  |  |  |
|  | a) | Report the assessment of the validity of the assumptions |  | The MR Egger intercept of all analyses was close to zero, indicating that horizontal pleiotropy did not significantly impact the MR results. |
|  | b) | Report any additional statistics (e.g., assessments of heterogeneity across genetic variants, such as *I^2^*, Q statistic or E-value) |  | Supplementary Table S7 |
| 13 | **Sensitivity analyses and additional analyses** |  |  |  |
|  | a) | Report any sensitivity analyses to assess the robustness of the main results to violations of the assumptions |  | In all analyses, the MR Egger intercept was close to zero, and the 95% CI was narrow. |
|  | b) | Report results from other sensitivity analyses or additional analyses |  | There was indication of SNP effect heterogeneity, but none of the analyses pinpointed specific SNPs as drivers of the causal relationship (Supplementary Figure S2). |
|  | c) | Report any assessment of direction of causal relationship (e.g., bidirectional MR) |  | Univariate positive MR analysis was hereby conducted to determine the protective effect of education level on MVPA deficiency. Higher education was found to be negatively correlated with lower MVPA (IVW: β=- 0.276,95%CI=-0.354 to-0.199, P=2.866×10-12).  The reverse MR analysis showed that MVPA did not affect educational attainment (IVW: P=0.165). |
|  | d) | When relevant, report and compare with estimates from non-MR analyses |  | NA |
|  | e) | Consider additional plots to visualize results (e.g., leave-one-out analyses) |  | However, leave-one-out analysis did not uncover any abnormal values (Supplementary Figure S3). |
|  | **DISCUSSION** |  |  |  |
| 14 | **Key results** | Summarize key results with reference to study objectives |  | Through the application of five complementary univariate MR methods with distinct basic assumptions, the findings consistently indicated a positive correlation between higher education levels and a reduced risk of MVPA. Moreover, the reverse MR analysis demonstrated that MVPA had no causal effect on education level. Furthermore, given the significant contrast in the causal effects estimated by univariate forward MR analysis and reverse MR analysis, a two-step MR analysis was further conducted. This approach revealed that the influence of education level on the risk of MVPA deficiency primarily stemmed from the protective effect of cognitive performance, with education level playing a partial role. |
| 15 | **Limitations** | Discuss limitations of the study, taking into account the validity of the IV assumptions, other sources of potential bias, and imprecision. Discuss both direction and magnitude of any potential bias and any efforts to address them |  | However, the research results still have some limitations to be considered when interpreting the results. |
| 16 | **Interpretation** |  |  |  |
|  | a) | Meaning: Give a cautious overall interpretation of results in the context of their limitations and in comparison with other studies |  | Our MR analyses identified a protective effect of genetically predicted educational attainment on MVPA risk. Our findings align with previously published research conclusions.  On the other hand, our current work also improves upon some previous studies. |
|  | b) | Mechanism: Discuss underlying biological mechanisms that could drive a potential causal relationship between the investigated exposure and the outcome, and whether the gene-environment equivalence assumption is reasonable. Use causal language carefully, clarifying that IV estimates may provide causal effects only under certain assumptions |  |  |
|  | c) | Clinical relevance: Discuss whether the results have clinical or public policy relevance, and to what extent they inform effect sizes of possible interventions |  | Therefore, it was hereby proposed that schools held an indisputable responsibility for fostering the healthy development of students.  It is vital for society to offer necessary support, including fostering a positive social sports atmosphere, providing ample sports opportunities, and ensuring top-notch sports infrastructure. |
| 17 | **Generalizability** | Discuss the generalizability of the study results (a) to other populations, (b) across other exposure periods/timings, and (c) across other levels of exposure |  | Finally, to address population stratification, the present analysis focused solely on individuals of European descent. However, further research involving other racial groups is still warranted to ensure broader applicability of the findings. |
|  | **OTHER INFORMATION** |  |  |  |
| 18 | **Funding** | Describe sources of funding and the role of funders in the present study and, if applicable, sources of funding for the databases and original study or studies on which the present study is based |  | Ministry of Education's Collaborative Education Initiative: An Exploration of Teaching Models for AR-Enhanced Aerobic Fitness Courses (Project Number: 202002064006) |
| 19 | **Data and data sharing** | Provide the data used to perform all analyses or report where and how the data can be accessed, and reference these sources in the article. Provide the statistical code needed to reproduce the results in the article, or report whether the code is publicly accessible and if so, where |  | The original data and code files were stored in the Open Science Framework (https://osf.io/crwah/). |
| 20 | **Conflicts of Interest** | All authors should declare all potential conflicts of interest |  | Disclosure of conflict of interest: The authors declare no conflicts of interest. |

This checklist is copyrighted by the Equator Network under the Creative Commons Attribution 3.0 Unported (CC BY 3.0) license.

1. **Skrivankova, V.W., Richmond, R.C., Woolf, B.A.R., Davies, N.M., Swanson, S.A., VanderWeele, T.J., Timpson, N.J., Higgins, J.P.T., Dimou, N., Langenberg, C., Loder, E.W., Golub, R.M., Egger, M., Davey Smith, G., and Richards, J.B. (2021a).** Strengthening the reporting of observational studies in epidemiology using mendelian randomisation (STROBE-MR): Explanation and elaboration. *BMJ* **375**,n2233. doi:10.1136/bmj.n2233

2. **Skrivankova, V.W., Richmond, R.C., Woolf, B.A.R., Yarmolinsky, J., Davies, N.M., Swanson, S.A., VanderWeele, T.J., Higgins, J.P.T., Timpson, N.J., Dimou, N., Langenberg, C., Golub, R.M., Loder, E.W., Gallo, V., Tybjaerg-Hansen, A., Davey Smith, G., Egger, M., and Richards, J.B. (2021b).** Strengthening the Reporting of Observational Studies in Epidemiology Using Mendelian Randomization: The STROBE-MR Statement. *JAMA* **326**,1614-1621. doi:10.1001/jama.2021.18236
